# The N-Terminal Domain of the *Drosophila* Retinoblastoma Protein Rbf1 Interacts with ORC and Associates with Chromatin in an E2F Independent Manner

**DOI:** 10.1371/journal.pone.0002831

**Published:** 2008-07-30

**Authors:** Joseph Ahlander, Xiao-Bo Chen, Giovanni Bosco

**Affiliations:** Department of Molecular and Cellular Biology, University of Arizona, Tucson, Arizona, United States of America; Centre National de la Recherche Scientifique, France

## Abstract

**Background:**

The retinoblastoma (Rb) tumor suppressor protein can function as a DNA replication inhibitor as well as a transcription factor. Regulation of DNA replication may occur through interaction of Rb with the origin recognition complex (ORC).

**Principal Findings:**

We characterized the interaction of *Drosophila* Rb, Rbf1, with ORC. Using expression of proteins in *Drosophila* S2 cells, we found that an N-terminal Rbf1 fragment (amino acids 1–345) is sufficient for Rbf1 association with ORC but does not bind to dE2F1. We also found that the C-terminal half of Rbf1 (amino acids 345–845) interacts with ORC. We observed that the amino-terminal domain of Rbf1 localizes to chromatin *in vivo* and associates with chromosomal regions implicated in replication initiation, including colocalization with Orc2 and acetylated histone H4.

**Conclusions/Significance:**

Our results suggest that Rbf1 can associate with ORC and chromatin through domains independent of the E2F binding site. We infer that Rbf1 may play a role in regulating replication directly through its association with ORC and/or chromatin factors other than E2F. Our data suggest an important role for retinoblastoma family proteins in cell proliferation and tumor suppression through interaction with the replication initiation machinery.

## Introduction

During the cell cycle each chromosome must be faithfully replicated before cell division. Numerous mechanisms exist to ensure the appropriate replication of chromosomes, including precise control of replication initiation [1], [2]. Limiting genomic DNA replication to just once per cell cycle ensures proper maintenance of gene dosage and ploidy, and failure to do so may lead to various pathologies, including cancer [3]–[5].

Specific locations in the genome, called origins of replication, are sites of DNA replication initiation during S phase. A heterohexameric protein complex called the origin recognition complex (ORC) binds to origins of replication and becomes a stage upon which the replication initiation machinery assembles. Cdc6 and Cdt1 associate with ORC and help recruit the MCM helicase complex. Many other factors are recruited, including DNA polymerase, which allow DNA replication to begin [1], [6]. Thus, the assembly of these proteins onto origins and regulation of their activities is a critical step in limiting DNA replication to once per cell cycle.

The retinoblastoma tumor suppressor (Rb) regulates DNA replication and is important for maintaining proper ploidy. Rb has been detected at sites of DNA replication [7]–[9]. It is required for S phase arrest in response to DNA damage, and *Rb* deficient cells can re-replicate their DNA to give polyploid cells [9]–14. Similarly, loss of the *Drosophila* Rb homologue, *rbf1*, results in inappropriate replication and mislocalization of Orc2 in follicle cells [15]. In addition, the temporal and spatial pattern of histone acetylation at a *Drosophila* replication origin is altered in *rbf1* mutant follicle cells, also suggesting a role for this protein in chromatin-mediated origin activity [16].

The canonical function of Rb is to restrict cell proliferation by binding and suppressing members of the E2F family of transcription factors, which results in downregulation of genes required for DNA synthesis and S phase progression [17], [18]. However, Rb also physically interacts with the proteins of many genes it transcriptionally regulates, such as MCM, DNA polymerase alpha, RFC, and Cyclin E [19]–[23]. Furthermore, human Rb can repress replication in a Xenopus cell-free and transcription-free system by binding to MCM [19], [24], [25]. Collectively, this evidence suggests that Rb may have a direct, post-transcriptional influence on DNA replication machinery. The molecular mechanisms through which Rb might directly influence origin activity are unclear.

The amino-terminal domain of Rb may play a role in regulating DNA replication initiation. Some *in vitro* replication assays have shown that the Rb amino-terminus can bind and inhibit MCM7, a component of the replicative helicase that is important for replication initiation and elongation [19], [25]. In this study we show that *Drosophila* Rbf1 interacts with ORC in an E2F independent manner through multiple domains that are outside of the E2F binding domain. The Rbf1 amino-terminal domain associates *in vivo* with chromosomal regions implicated in replication initiation, including colocalization with Orc2 and acetylated histone H4. Significantly, our work illustrates novel interactions of Rb with the replication initiation machinery that have important implications for our understanding of cell proliferation and tumor suppression.

## Results

### Rbf1 interacts with ORC through multiple domains

**Figure 1:**
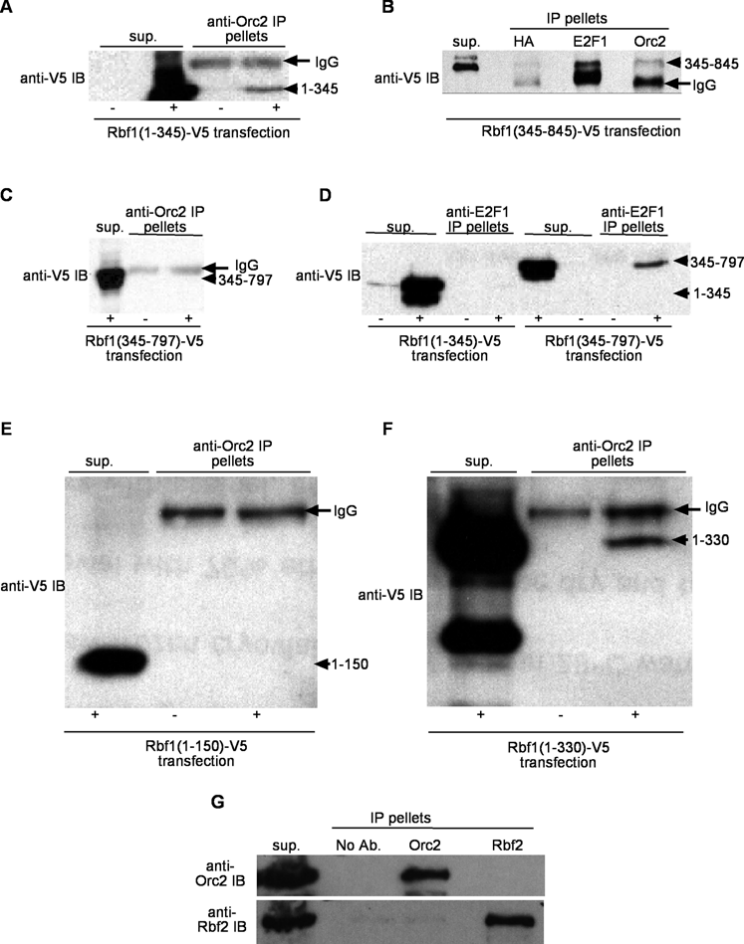
ORC interacts with Rbf1 N-terminal and C-terminal fragments in an E2F independent manner. S2 cells were transfected with metallothionein promoter (pMT) regulated Rbf1 deletion constructs with a C-terminal Simian Virus 5 (V5) epitope-tag and cell extracts from uninduced (−) and copper sulfate induced (+) cultures were subjected to immunoprecipitation (IP) and anti-V5 immunoblotting (IB). (A) Rbf1(1–345)-V5 transfected cell extracts were immunoprecipitated with anti-Orc2. Note that extracts from induced cells show a Rbf1(1–345)-V5 fragment (arrowhead) in the IP pellet while uninduced cell extracts treated identically with anti-Orc2 serum failed to IP an anti-V5 reacting band of comparable size. (B) Rbf1(345–845)-V5 transfected cell extracts were induced and anti-HA (negative control), anti-dE2F1 and anti-Orc2 serum were used in IP reactions. Western IB was probed with anti-V5 which detects the Rbf1(345–845)-V5 protein fragment (arrowhead) that migrates just above the IgG heavy chain (arrow). (C) Extracts from Rbf1(345–797)-V5 cells uninduced (−) and induced (+) cultures were subjected to anti-Orc2 IP and western IB probed with anti-V5. (D) Extracts from Rbf1(1–345)-V5 or Rbf1(345–797)-V5 cells uninduced (−) and induced (+) were subjected to anti-dE2F1 IP. Extracts from (E) Rbf1(1–150)-V5 and (F) Rbf1(1–330)-V5 cells that were uninduced (−) and induced (+) were subjected to anti-Orc2 IP and anti-V5 western IB. In each case 5–10% of the IP supernatant (sup.) and all of the IP pellets were loaded. In all panels (except D) the IgG heavy chain protein is noted by an arrow and Rbf1-V5 deletion fragments are denoted by an arrowhead. (G) Rbf2 does not interact with ORC. Ovarian extracts were immunoprecipitated (IP pellets) with no antibody (No Ab.), anti-Orc2 or anti-Rbf2. Entire IP pellets and 10% of supernatant were loaded. Immunoblot (IB) was first probed with anti-Orc2, stripped and then reprobed with anti-Rbf2.

We showed previously by coimmunoprecipitation that *Drosophila* Orc1 and Orc2 proteins interact with the dDP/dE2F1/Rbf1 complex in ovarian extracts, and we wished to further characterize this interaction [15]. Previous experiments using *e2f1^i2^* mutant flies [26] demonstrated that ORC does not interact with a truncated dE2F1 that has lost its Rbf1 interaction domain, which suggested that Rbf1 might mediate the ORC-dE2F interaction [15]. We used *Drosophila* S2 cell culture to test the association of transiently transfected Rbf1 proteins with endogenous ORC proteins. We expressed V5 epitope tagged Rbf1 deletion fragments in S2 cells under the inducible metallothionein promoter [27] and tested whether they would coimmunoprecipitate with endogenous Orc2. We found that the Rbf1 N-terminal fragment (Rbf1N, amino acids 1–345) was sufficient for its interaction with ORC (Figure 1A). We next tested the Rbf1-ORC interaction in S2 cells using two different constructs of the Rbf1 C-terminal fragment, one with amino acids 345–845 and another with amino acids 345–797. We observed that the Rbf1(345–845)-V5 fragment could be coimmunoprecipitated with Orc2 (Figure 1B). Interestingly, the Rbf1(345–797)-V5 fragment did not pellet with Orc2 immunoprecipitates (Figure 1C). This data suggests that an interaction of ORC with the Rbf1 C-terminus requires amino acids 797–845 of Rbf1. However, it was also possible that the 345–797 fragment could not properly fold into a functional protein. It has been shown previously that the C-terminal half of Rbf1 contains a pocket domain that interacts with dE2F and that the 345–797 fragment is sufficient for this interaction [28]. Therefore, we asked whether the V5 tagged Rbf1 345–797 fragment could still associate with dE2F1 in S2 cell extracts. Indeed, Rbf1(345–797)-V5 was found in dE2F1 immunoprecipitates while Rbf1(1–345)-V5 was not (Figure 1D). Additional deletions of the Rbf1 N-terminal region into amino acids 1–150 (Figure 1E) and amino acids 1–330 (Figure 1F) allowed us to further define the N-terminal 150–330 Rbf1 amino acids as being necessary for its association with Orc2. These observations indicate that ORC interacts with Rbf1 through multiple sites distinct from the dE2F binding site. Since there are two Rb family genes in *Drosophila*, Rbf1 and Rbf2, we tested whether ORC also interacts with Rbf2. We had previously shown that endogenous Rbf1 from *Drosophila* ovarian extracts interacts with ORC [15]. Intriguingly, endogenous Orc2 and Rbf2 could not be coimmunoprecipitated from ovarian extracts (Figure 1G), suggesting that ORC interacts specifically with Rbf1 and not Rbf2.

### Nuclear localization and chromatin association of Rbf1N

**Figure 2:**
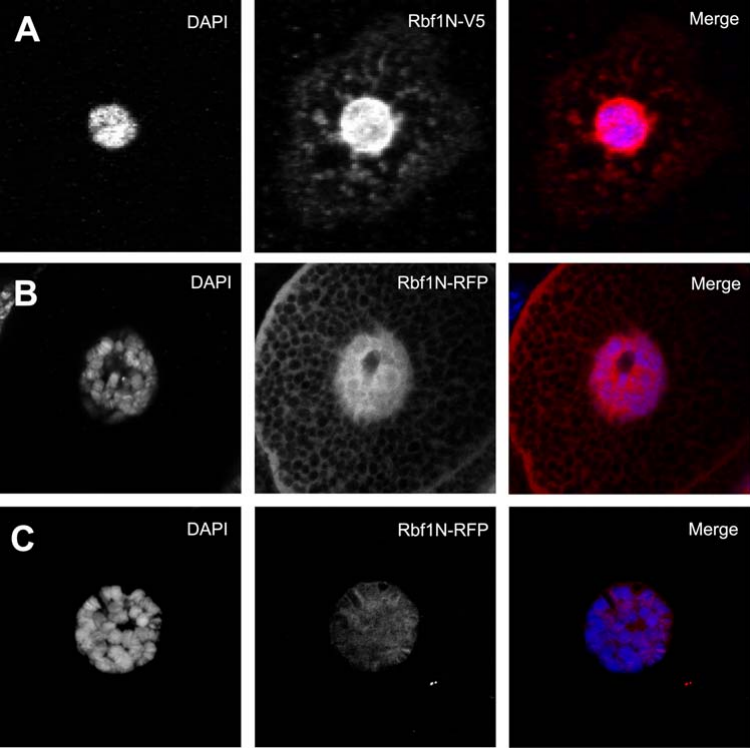
The Rbf1 amino-terminal domain, Rbf1N, is sufficient for nuclear localization and chromatin association. (A) S2 cells were transfected with a copper inducible construct containing Rbf1N (Rbf1 amino acids 1–345) tagged with a V5 epitope. Immunofluorescence using V5 antibodies shows Rbf1N is mostly nuclear with small amounts cytoplasmic localization. To observe the localization of Rbf1N in vivo, transgenic flies containing UAS>Rbf1N-RFP were crossed to flies bearing a GAL4 transgene that expressed specifically in salivary glands. (B) Rbf1N-RFP localizes to the nucleus in salivary gland cells. RFP fluorescence is brightly seen throughout the nucleus and cytoplasm, and it appears to also associate with cytoplasmic structures and the plasma membrane. (C) To remove unbound Rbf1N-RFP, salivary glands were incubated in chromatin wash buffer, revealing that Rbf1N-RFP associates with chromatin and localizes in a striped pattern along polytene chromosomes.

It was previously shown that the amino-terminus of human Rb alone cannot localize to the nucleus without an added nuclear localization signal [29]. However, data presented above suggested that *Drosophila* Rbf1 might be tethered to chromatin independently of dE2F by association with other nuclear proteins. To examine the intracellular localization of the amino-terminus of *Drosophila* Rbf1, we transfected S2 cells with Rbf1(1–345)-V5, hereafter referred to as Rbf1N. Immunostaining of V5 shows Rbf1N localizes strongly to the nucleus, with some cytoplasmic staining (Figure 2A).

To further study the localization of Rbf1N *in vivo*, we made transgenic flies with Rbf1N-V5 fused to the mCherry red fluorescent protein [30] in the pUASP expression vector [31]. Expression of Rbf1N-RFP using tissue-specific GAL4 drivers shows robust nuclear localization in larval salivary gland cells in addition to cytoplasmic and plasma membrane localization (Figure 2B). Furthermore, treatment with chromatin wash buffer before fixation [32] reveals that Rbf1N is chromatin-associated (Figure 2C). It may be that some of the recruitment of Rbf1N to chromatin is due to its association with ORC, although Rbf1N is probably recruited to many other sites through its interaction with other nuclear proteins, such as MCM (data not shown). Expression of Rbf1N in ovarian nurse cells and follicle cells also exhibited nuclear localization (data not shown). Thus, the amino-terminal domain of Rbf1 is sufficient for nuclear localization and chromatin association *in vivo* in a variety of cell types.

### Rbf1N colocalizes with acetyl-H4 at interbands of polytene chromosomes

**Figure 3:**
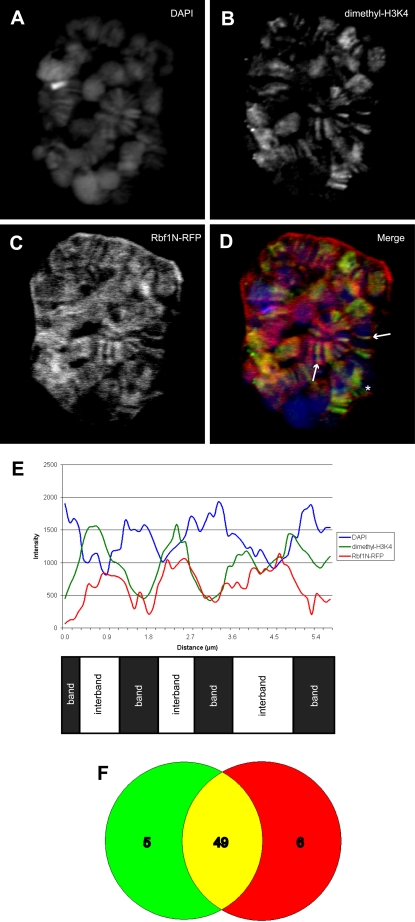
Rbf1N colocalizes with modified histones at interband regions of salivary gland polytene chromosomes. (A) Salivary glands expressing Rbf1N-RFP were chromatin washed and counterstained with antibodies specific for histone H3 dimetylated on lysine 4, a modified histone that marks interband DNA and is an indicator of active transcription. Rbf1N-RFP (C) and dimethyl-H3K4 (B) colocalize at interbands (D and E), whereas DAPI stains the bands of the polytene chromosomes (A and E). Arrows indicate interbands demonstrating colocalization, and the asterisk denotes a site where colocalization does not occur. The merged image (D) reveals extensive colocalization of Rbf1N-RFP and dimethyl-H3K4, as well as some areas of non-overlap. (E) A graph of fluorescent intensity along several chromosome bands shows the banding pattern of DAPI versus the alternating interband pattern of Rbf1N-RFP and dimethyl-H3K4. (F) A Venn diagram illustrates that Rbf1N-RFP colocalizes extensively with the modified histone dimethyl-H3K4 in randomly chosen bands.

**Figure 4:**
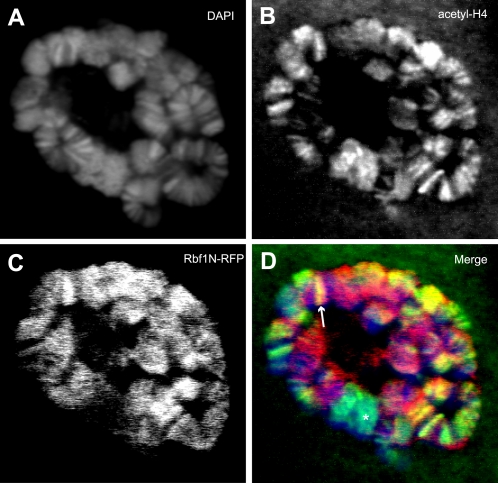
Rbf1N colocalizes with acetylated histone H4 at interband regions of salivary gland polytene chromosomes. Salivary glands expressing Rbf1N-RFP were chromatin washed and counterstained with antibodies specific for acetylated histone H4, a marker of active transcription and active origins of replication. Acetyl-H4 colocalizes with Rbf1N-RFP at interbands. (A) DAPI staining marks chromosomal bands. Acetyl-H4 (B) and Rbf1N-RFP (C) colocalize at many chromosomal locations (D). The merged image (D) reveals extensive colocalization of Rbf1N-RFP and acetyl-H4, as well as some areas of non-overlap. The arrow indicates an interband representing colocalization, and the asterisk denotes a site where colocalization does not occur.

We next wished to understand the character of the chromatin with which Rbf1N is associated in order to gain insight into its function. We expressed Rbf1N-RFP in larvae using GAL4 drivers expressed specifically in the salivary glands. The salivary glands were incubated in chromatin wash buffer before being fixed with formaldehyde to remove any unbound Rbf1N-RFP. Confocal images of whole-mount nuclei reveal that Rbf1N-RFP localizes specifically to the regions in between DNA bands stained by DAPI, called interband DNA (Figure 3). Co-staining with an antibody directed against the modified histone dimethyl-H3K4, a marker of interbands [33], confirms that Rbf1N-RFP is enriched at interbands. In addition, measurements of fluorescent intensity along chromosome bands visibly exhibit the interband localization of Rbf1N-RFP and dimethyl-H3K4 in between the DAPI bands (Figure 3E). Rbf1N-RFP appears to be more broadly distributed across the chromosomes than dimethyl-H3K4. However, a closer analysis of colocalization revealed that 49 out of 54 dimethyl-H3K4 bands chosen at random overlap conspicuously with Rbf1N-RFP (Figure 3F). Interestingly, Rbf1N-RFP localization on polytene chromosomes also overlaps consistently with acetylated histone H4 (Figure 4), a histone modification that was shown to also mark active origins of replication in *Drosophila* follicle cells [16], [34]. Both of these histone markers indicate that Rbf1N is highly enriched at chromatin regions involved in active transcription and/or DNA replication. We note that there some regions of dimethyl-H3K4 and acetyl-H4 enrichment where Rbf1N-RFP is not (Figure 3D and Figure 4D, asterisks). These may be specific regions where Rbf1N is not recruited, and thus would not have a role in altering local activity at these sites. Our results indicate that the amino-terminal domain of Rbf1 is sufficient to localize to interband DNA of polytene chromosomes at regions of active chromatin, and may therefore play a role in modulating transcription and/or DNA replication at these sites where it is recruited.

### Chromatin-associated Rbf1N colocalizes with ORC *in vivo*

**Figure 5:**
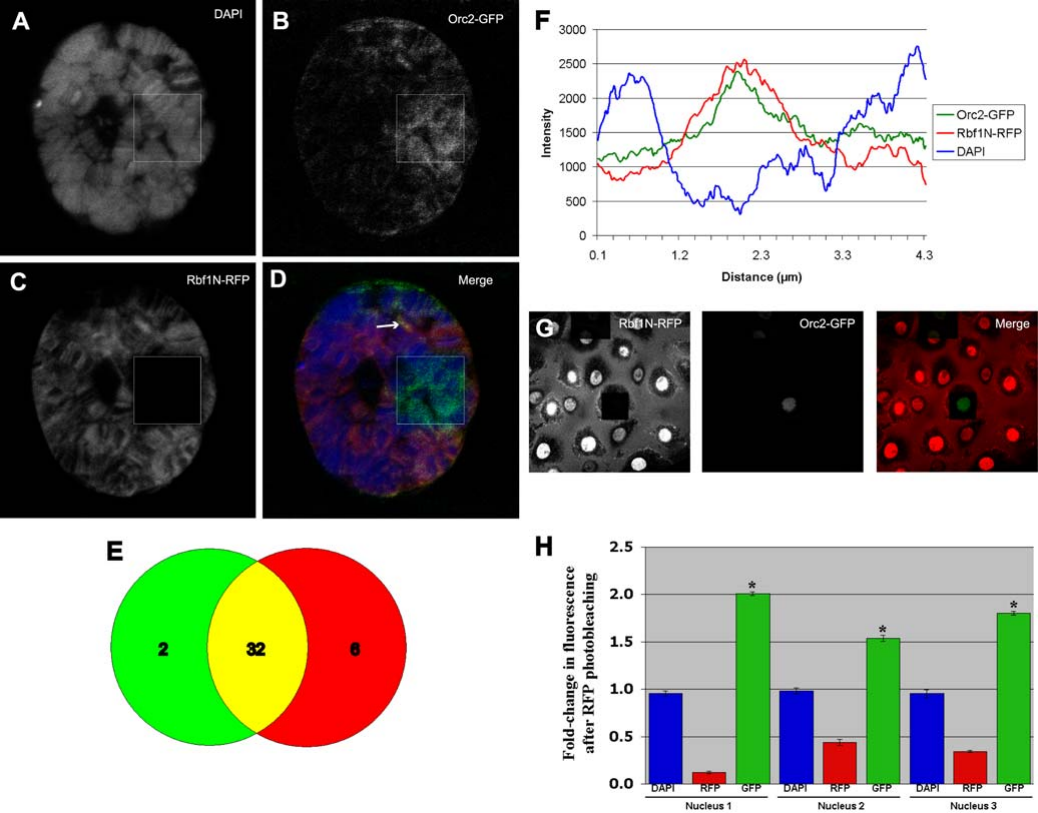
Rbf1N physically interacts with ORC *in vivo*. Salivary glands from transgenic larvae expressing both Rbf1N-RFP and Orc2-GFP were chromatin washed and fixed for fluorescence microscopy. Rbf1N-RFP (C) and Orc2-GFP (B) colocalize on polytene chromosomes (D through F). DAPI stains the bands of the polytene chromosomes (A). Photobleaching of Rbf1N-RFP, indicated by the boxed area, results in an increased GFP signal, which is a consequence of fluorescence resonance energy transfer (FRET) by the red and green fluorescent proteins, mCherry and EGFP. FRET reveals that Rbf1N-RFP and Orc2-GFP are in very close physical proximity. (E) A Venn diagram illustrates that Orc2-GFP colocalizes extensively with Rbf1N-RFP fluorescence in randomly chosen bands. (F) A graph of fluorescent intensity along the region indicated by an arrow (D) shows that Rbf1N-RFP and Orc2-GFP colocalize within an interband region. (G) Photobleaching of RbfN-RFP results in an increased GFP signal in salivary gland nuclei. (H) Fold-change after Rbf1-N-RFP photobleaching is shown as the ratio of bleached/non-bleached signal in each of three different nuclei. Blue bars show DAPI signal, red is RFP signal and green show fold-change in Orc2-GFP signal. A two-tailed T-test indicates that GFP fluorescence increase is highly statistically significant p<0.0001 in each of the three nuclei. RFP photobleaching increases GFP fluorescence by 1.5–2-fold. These three nuclei (see Figure S1) are representative of larger populations.

We next determined whether chromatin-associated Rbf1N interacts with ORC *in vivo*. We collected flies containing both transgenes *Sgs3>GAL4* and *UAS>Rbf1N-RFP* and crossed them to Orc2-GFP flies, which have an engineered exon containing the EGFP coding sequence inserted into the coding region of endogenous *Orc2* [35]. We dissected salivary glands from wandering third instar larvae and incubated them in chromatin wash buffer before fixation. Rbf1N-RFP fluoresced strongly (Figure 5C), as expected, whereas the Orc2-GFP fluorescence was generally faint (Figure 5B). Rbf1N and Orc2 appear to colocalize in many places on the chromosomes (Figure 5D), where 32 out of 40 bands chosen at random contain both Rbf1N-RFP and Orc2-GFP (Figure 5E). Measurement of fluorescent intensity along one region of the chromosome shows that colocalization of Rbf1N and Orc2 occurs in an interband (Figure 5F). It is also interesting to note that photobleaching of Rbf1N-RFP resulted in a modest increase in GFP signal (Figure 5D boxed area, and Figure 5G), which may be an indication of FRET. Previous studies illustrated that EGFP and mCherry have the ability to exhibit fluorescence resonance energy transfer (FRET) with a Förster radius at 5.4nm, which is the distance at which 50% of the excited EGFP molecules are neutralized by FRET [36], [37]. Thus, it appears that Rbf1N-RFP complexes with ORC and is within sufficient proximity (1–10 nm) so as to neutralize some of the light emission from Orc2-GFP, which is characteristic of FRET. Upon photobleaching, Rbf1N-RFP can no longer absorb the GFP emission, which allows us to more fully visualize the Orc2-GFP. To quantify FRET we photobleached RFP in a discrete section of three different nuclei and compared the amount of fluorescence between photobleached and non-photobleached areas within the same nucleus. We found that GFP fluorescence increases 1.5–2 fold after RFP photobleaching (Figure 5H). A two-tailed T-test indicates that GFP fluorescence increase is highly statistically significant p<0.0001 in each of the three nuclei. Consistent with our immunoprecipitation data (Figure 1), we conclude that Rbf1N and Orc2 colocalize on polytene chromosomes.

### The N-terminal domain of Rbf1 is not sufficient for altering cell cycle or DNA replication

Given the the *in vitro* interactions of Rbf1N with ORC and colocalization of Rbf1N and Orc2, we hypothesized that the Rbf1N domain may function to regulate cell cycle progression in general and DNA replication in particular. To test this hypothesis we overexpressed the Rbf1N protein in tissues of transgenic *Drosophila*. First, *actin>GAL4* driving expression of the *UAS>Rbf1N-RFP* was examined in the ovarian follicle cells. We observed robust expression and nuclear localization of the Rbf1N-RFP in follicle cells, however BrdU labeling of follicle cells did not reveal any detectable changes in DNA replication patterns during endoreplication or chorion gene amplification (data not shown). Flow cytometry analysis of follicle cell nuclei also did not reveal any significant differences in ploidy content (Table S1), proportion of follicle cells in S phase (Table S2) or nuclear size (Table S3) versus controls. In addition, overexpression of Rbf1N-RFP in diploid proliferating neuroblasts similarly did not cause any cell cycle pertubation, as assayed by flow cytometry (data not shown). Lastly, overexpression of different independent insertion lines of *UAS>Rbf1N-RFP* in the developing eye by *GMR>GAL4* [38] or *ey>GAL4* [39] did not yield any obvious adult eye phenotypes (data not shown). These data suggest that although Rbf1N is sufficient for chromatin localization and its interaction with Orc2, the Rbf1N (1–345) domain alone is not sufficient for significantly altering the cell cycle *in vivo*.

### Rbf1 has a conserved tandem cyclin fold structure

Despite our failure to detect an *in vivo* phenotype when overexpressing the Rbf1N fragment it may be that this region of Rbf1 is nevertheless critical for a multitude of cellular functions. Although there is an abundance of information regarding the function of the C-terminal pocket domain of Rb, there is little known about the function of the amino-terminal domain of Rb family members [40]. We analyzed the *Drosophila* retinoblastoma proteins to determine the extent of sequence and structure conservation of the N-terminal domain between flies and humans. Our analysis using protein sequence alignments reveals that the N-terminal domain of Rbf1 is highly conserved within Drosophilidae as well as between flies and humans (Figure S2). Such protein sequence conservation supports our hypothesis that the N-terminal region of Rbf1 may have important *in vivo* functions and that further analysis of this domain is warranted.

**Figure 6:**
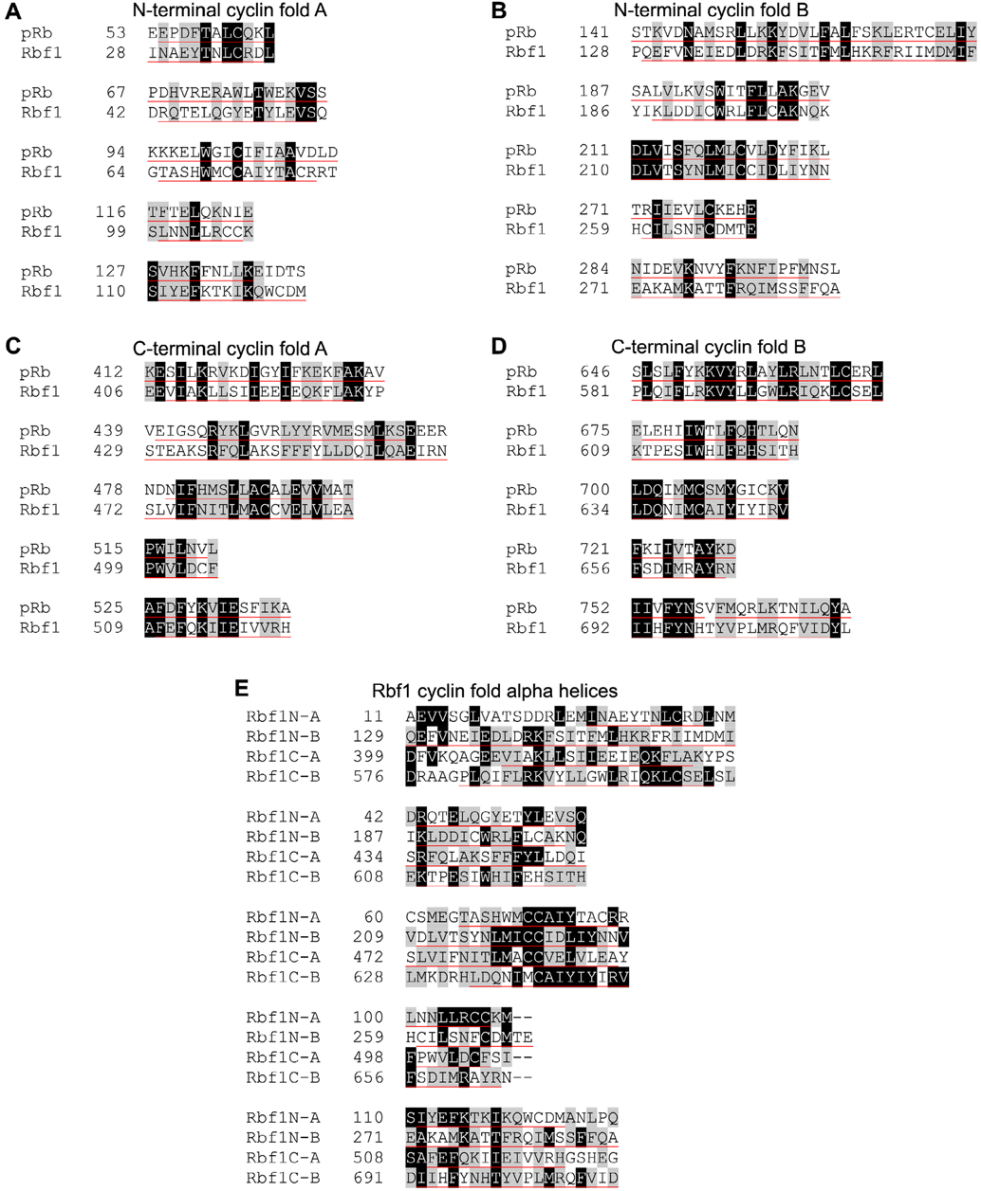
Alignment of Cyclin fold helices within the Rbf1 sequence. The retinoblastoma proteins in humans and flies share a domain structure containing four cyclin folds, with each fold consisting of five alpha helices. The N-terminal (A and B) and C-terminal (C and D) domains of Rbf1 each have a cyclin fold A and B, resulting in four total cyclin folds that share extensive sequence conservation with pRb. It is likely that the retinoblastoma family of proteins emerged from two successive tandem duplication events from an ancient cyclin-like ancestor that gave rise to many cell cycle regulators. This finding seems to indicate that the retinoblastoma N and C-terminal domains are intrahomologues. The tandem domain architecture of Rb family proteins may explain our finding that ORC interacts with multiple Rbf1 domains, and suggests that Rbf1 may be an adaptor molecule that is able to switch between several orientations with ORC to accommodate different combinations of binding partners depending on different cellular contexts. (E) All five helices from the four Rbf1 cyclin folds were compared together. Amino acids conserved in two or more helices were shaded accordingly, revealing a collective conservation of amino acid sequence between the cyclin folds. Black shading with white letters indicates identical amino acids. Grey shading indicates amino acid similarity. Helices are underlined in red.

**Figure 7:**
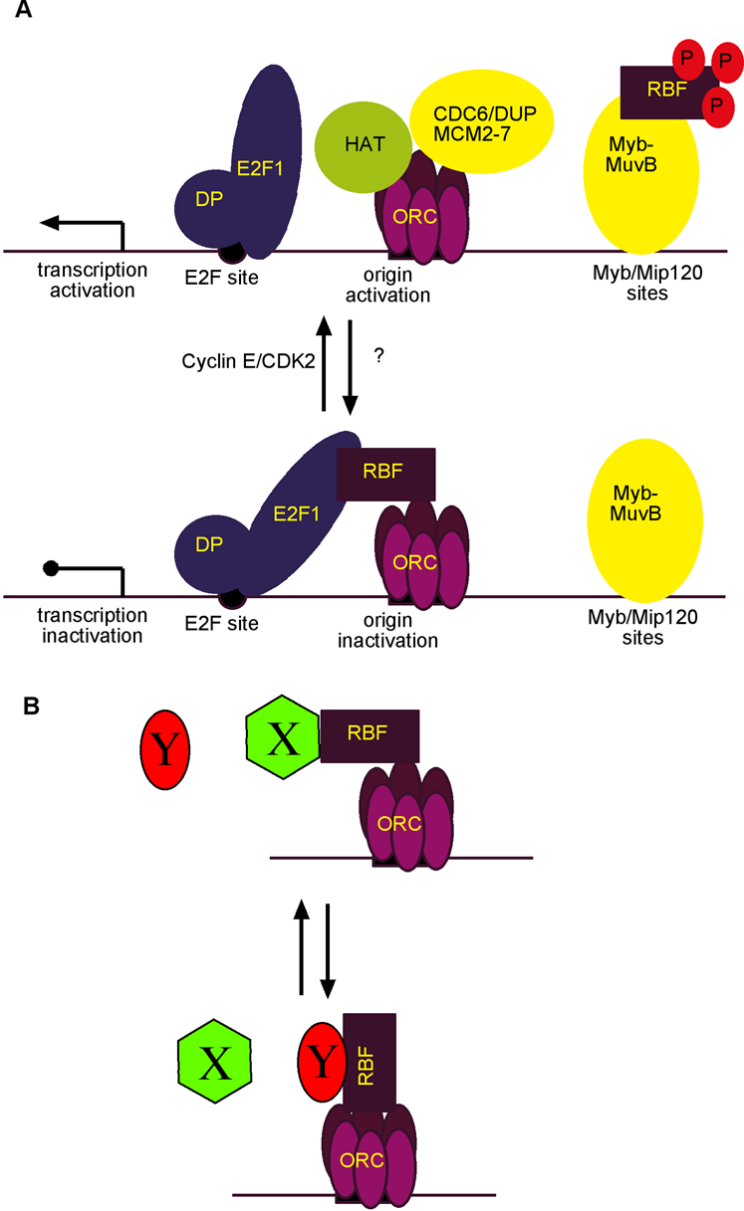
Models of Rbf1 adaptor functions. (A) Rbf1 associates with ORC and may inhibit recruitment other replication initiation factors. Due to its association with ORC, Rbf1 might inhibit the activity of the replication initiation complex. Phosphorylation of the C-terminal domain of Rbf1 by Cyclin-CDK complexes releases binding partners, such as E2F, and may constitute part of a reversible switch to regulate origins of replication. This switchable regulation may come in part through changes in recruitment of associated chromatin modifying enzymes and tethering of phosphorylated Rbf1 by the Myb-MuvB complex may allow Rbf1 to ping-pong from one complex to another in a localized manner. (B) We speculate that because Rbf1 may be able to associate with chromatin bound ORC and through multiple domains it can be tethered in more than one orientation, thereby presenting and/or occluding docking sites for other Rbf1-associated chromatin factors (e.g. histone deacetylases, histone methyltransferase, etc.). For example, this may allow Rbf1 to function as an “adaptor” molecule at any one ORC site where its specific orientation dictates which factors (depicted as “X” and “Y”) may or may not be present at any given time. This model predicts that a single genomic site may have constitutive ORC/Rbf1 localization while re-orientation of the Rbf1 molecule can mediate the recruitment of different suites of chromatin modifying enzymes. This model and that described above (A) are not mutually exclusive.

We also explored a structural analysis of Rbf1N. Protein fold analysis using Phyre [41], [42] showed that Rbf1N contains a cyclin-like fold with high similarity to transcription factor TFIIb, and this was also true for human pRb, p107, and p130. Using amino acid sequence alignment with pRb guided by secondary structure prediction of Rbf1, we observed that each Rbf1 domain contains tandem cyclin folds consisting of five alpha helices each (Figure 6). Previous studies using structural analysis have indicated that human pRb contains tandem cyclin-like folds in both its N and C-terminal domains, and suggesting that this family of proteins emerged from two successive tandem duplication events possibly sharing an ancient common ancestor that gave rise to multiple cell cycle regulators [43], [44]. Moreover, since the alpha helices comprising each of the N-terminal and C-terminal Cyclin folds of Rbf1 share sequence similarity (Figure 6E), this intrahomologous tandem domain architecture of retinoblastoma proteins may explain our finding that ORC interacts with multiple Rbf1 domains (Figure 1). Rbf1 may be an adaptor molecule that is able to switch between several orientations with ORC to accommodate different combinations of binding partners depending on the cellular context (Figure 7B). Such high conservation of both sequence and structure of the retinoblastoma N-terminal domain will provide the basis for future studies using directed mutagenesis for *in vitro* and genetic functional studies.

## Discussion

The retinoblastoma tumor suppressor, Rb, plays a significant role in regulating the cell cycle, including S phase [18]. *Rb* deficient cells in both flies and mammals show a reduced ability to restrict re-replication of DNA [13]–[15], which may lead to genome instability and tumor progression [5]. It is clear that Rb negatively regulates DNA replication indirectly by shutting down gene expression of crucial replication factors [17], [45], [46]. However, it remains to be seen how much Rb directly influences the replication machinery itself. In this study we present evidence that *Drosophila* Rbf1 associates with ORC through multiple domains, further supporting a role for Rbf1 in regulating DNA replication.

Our immunoprecipitation data demonstrate that ORC interacts with Rbf1 independent of dE2F1 binding. ORC interacts with the N-terminal domain of Rbf1 (Figure 1A), whereas E2F interacts only with the C-terminal pocket containing region (Figure 1D). We also show that ORC has a second interaction site on the C-terminal domain of Rbf1 that appears to require a region outside of the E2F binding domain on Rbf1 (Figure 1B and 1C). Previous studies have identified a number of mammalian Rb binding proteins that also interact with both the N- and C-terminal domains [43], [47]–[50]. Our finding that Rbf1 can interact with chromosomal proteins like ORC regardless of E2F association gives fresh insight into the tumor suppressive properties of retinoblastoma proteins, since they may retain the potential to regulate cellular events, such as replication initiation, even while E2F binding to RB is inhibited by mutation, phosphorylation, or binding of viral oncoproteins [18].

We show that the Rbf1 amino-terminal domain, Rbf1N, is sufficient for nuclear localization and chromatin association *in vivo*. Significantly, we show that Rbf1N localizes to interband regions on larval salivary gland polytene chromosomes (Figure 3). *Drosophila* polytene chromosomes have long served as a model for studying genetics and chromatin dynamics for [51], and several studies have highlighted their potential in studying the properties of replication timing along the chromosome [52], [53]. A comparison of the characteristics of interbands of salivary gland polytene chromosomes and early origins of replication in Kc cell culture reveal striking similarities. Interbands and early origins are both AT rich, are enriched with RNA polymerase II, and are transcriptionally active [54]–[56]. Furthermore, they are enriched with ORC, incorporate BrdU, and replicate early in S phase [52], [57], [58]. These previously published observations suggest that interbands may contain origins of replication. Our results further support an interband origin hypothesis. First, we show that Rbf1N localizes to interbands (Figure 3). Second, Rbf1N colocalizes at interbands with acetylated histone H4 (Figure 4), a histone modification that has been shown to be associated with active origins of replication in *Drosophila* amplification stage follicle cells [16], [34]. Third, we show that Rbf1N colocalizes with Orc2 at interbands (Figure 5). Collectively, these observations support a hypothesis in which interbands serve as places for assembly of replication initiation complexes, including ORC and Rbf1. Since we were not able to demonstrate that *in vivo* expression of the Rbf1-N domain alone was sufficient to perturb replication and cell cycle progression, further studies will be required to characterize its function and the chromosomal sites bound specifically by the ORC-Rbf1 complex.

Many lines of genetic and biochemical evidence suggest that Rb restricts replication initiation, although its mechanism is not clearly understood. We suggest a model describing how Rbf1 might directly regulate replication initiation (Figure 7A). First, Rbf1 binding may inhibit ORC complex formation with other replication initiation factors. Second, Rbf1 may inhibit activity of the replication initiation machinery after it has assembled at an origin of replication. Third, Rbf1 may recruit chromatin modifying factors to origins of replication to suppress origin activity before and/or after replication initiation. For example, retinoblastoma family proteins associate with histone methyl transferases and histone deacetylases [59], [60]. Histone acetylation status has been shown to correlate with origin activity in *Drosophila* [16], [34]. In addition, *rbf1* mutant follicle cells have overactive origins associated with prolonged H4 acetylation [15], [16]. Rbf1 may be employed in ways such as these to inhibit premature origin firing or, perhaps more importantly, prevent reinitiation of DNA replication during the cell cycle.

The amino-terminal domain of Rbf1 is sufficient for the interaction with ORC. However, the Rbf1 N-terminus alone may not be sufficient for inhibition of DNA replication initiation *in vivo* and thus may serve to recruit other important factors, such as histone modifying enzymes, to origins of replication through the C-terminal domain (Figure 7). Furthermore, this N-terminal tether to ORC may retain Rbf1 at origins of replication throughout the cell cycle even while the repertoire of binding partners changes on its C-terminus. For example, there are a few putative CDK phosphorylation sites on Rbf1, but all of them reside within the C-terminal pocket domain [28]. Phosphorylation by cyclin-CDKs dissociates Rbf1-dE2F1 complexes [61]. However, phosphorylation of Rbf1 by Cyclin E-cdk2 is not sufficient to prevent its association with the Myb-MuvB complex [61]. In another study, human Rb was shown to associate with chromatin well into S-phase at a time when its phosphorylation status typically prevents its association with E2F [62]. These findings raise the interesting possibility that cell cycle mediated phosphorylation of Rbf1 can modulate protein interactions while Rbf1 remains tethered to specific chromosomal sites, such as origins of replication through its association with ORC and/or Myb-MuvB. In addition, Rbf1 may also associate with other replication factors, for example RFC and MCM complexes that may also serve to tether Rbf1 to chromatin. Our observations and data reported by others support a model in which Rbf1 may constitute part of a sensor switch at origins of replication and/or sites of transcription that can be rapidly disabled to allow for replication initiation or gene transcription, while its physical presence allows it to be reactivated just as quickly to repress these processes in response to specific cues, such as DNA damage or developmental signals [16], [34], [61], [63]–[65]. Cell cycle and mutational analysis of the ORC-Rbf1 interaction will give us more insight into the mechanism of Rbf1 at origins of replication.

Retinoblastoma proteins are generally thought to be recruited to chromatin *in vivo* through DNA binding proteins, such as E2F, although a limited number of studies show some ability of Rb to bind nonspecifically to DNA *in vitro* [66], [67]. Our observations that Rbf1 can associate with ORC independently of E2F raises the possibility that Rbf1 could be tethered to chromatin and act as a transcriptional regulator for genes that do not contain E2F binding sites [68] (Figure 7B). ORC has been implicated in transcriptional roles, as well [69]. Moreover, transcriptional activity and DNA replication timing appear to be tightly coordinated at a local chromatin level [52], [53], which suggests that the transcription repressor functions of Rbf1 may be co-opted to also regulate replication initiation. Rbf1 may possibly be recruited to interband regions of polytene chromosomes by both its association with replication factors and its association with the basal transcription machinery [70]. The coincidence of both replicative and transcriptional components at polytene interbands may signify a dual role of Rbf1 in these processes.

Given that the amino-terminal domain of Rb family members is conserved between flies and mammals (Figure 6 and Figure S2), it is astonishing that this domain is largely ignored in the experimental literature [40]. In fact, many publications have characterized Rb protein interaction and function using only N-terminally deleted pRb constructs. Notwithstanding, of the handful of reports that have explored the function of the Rb N-terminal domain, two have shown that it may play a role in suppression of apoptosis and tumor formation [71], [72]. In addition, the amino-terminal domain of p107 is necessary for growth inhibition and can bind and inhibit cyclin-CDK complexes [73]. The tandem duplication of cyclin folds in both Rb domains [43], [44] that is conserved in Rbf1 (Figure 6) may explain how Rbf1 associates with the same complexes (i.e. ORC) through multiple domains. Consequently, it is interesting to speculate that Rbf1 can be tethered to chromatin by a single complex (e.g. ORC) in different orientations (Figure 7B). This idea adds further complexity to the sensor switch model in that any given genomic locus where Rbf1 is tethered may have very different chromatin states (at different times or in different cells) that are determined as Rbf1 “rotates” through its multiple binding sites with its tether (Figure 7B). The “rotation” or “ping-pong” models [16] predict that the Rbf1 binding orientation would occlude or present additional docking sites on Rbf1 for factors that can only associate through single and specific sites on the Rbf1 protein. Although speculative, this model is consistent with the observations presented in this study as well as previous reports on Rbf1 function [16], [34], [61], [63]–[65].

We have presented data using the N-terminus of Rbf1 that suggest an important and conserved role for retinoblastoma family proteins in cell proliferation and tumor suppression through interaction with the replication initiation machinery. Although we have failed to observe any appreciable cell cycle function of the Rbf1N domain by itself, we nevertheless propose that this domain plays an important function by creating multiple protein binding configurations and by tethering Rbf1 to chromatin. Protein sequence and structural conservation between humans and flies and within Drosophilidae also suggests a conserved and unappreciated function of the N-terminal domain of retinoblastoma tumor suppressor proteins. We speculate that the amino-terminal domain of Rb in both flies and humans has much to reveal about cell cycle control and cancer biology that merits further investigation.

## Materials and Methods

### RBF1 expression constructs and transgenic flies

All C-terminal V5-tagged RBF1 proteins were expressed under the metallothionein gene promoter [27] in the *Drosophila* pMT/V5-HisB expression plasmid (Invitrogen). RBF1 cDNA fragments [74] were PCR amplified with KpnI and SacII restriction sites designed into the 5′-primer and 3′-primer, respectively, for all V5-tagged proteins. For RBF1 1–345 (Rbf1N) the primers used were 5′-CTTGGTACCTATGAGCGAGCCTGACCCGCAG-3′ and 5′-TCCTCCCCGCGGGGCAGTGTGTTCCCCCGCATC-3′. For RBF1 345–797 (Rbf1C) the primers used were 5′-CTTGGTACCTATGGCCCTCAACGACCAGTCCCTG-3′ and 5′-TCCTCCCCGCGGCTAGTCCGGCTCGTCGCCAAAGCT-3′. Subsequent restriction digestion and cloning of PCR products was done directly into the vector. All clones were validated by sequencing. To generate the UAS>Rbf1N-RFP vector, the mCherry RFP [30] coding sequence was PCR amplified with primers designed with 5′ SpeI and 3′ XbaI sites: 5′-ATAGTAGTATGGTGAGCAAGGGCGAG-3′ and 5′-GCTCTAGATTACTTGTACAGCTCGTCCAT-3′. The mCherry PCR product was digested and cloned into pUASP [31]. Subsequently, we used the RBF1-containing pMT/V5-HisB constructs described above to PCR amplify RBF1 amino acids 1–345 with the V5 epitope tag with primers designed with 5′KpnI and 3′ SpeI sites: 5′-ATAGGTACCATGAGCGAGCCTGACCCGCA-3′ and 5′-CGCACTAGTCGTAGAATCGAGACCGAGGA-3′. This PCR product was digested and subcloned in-frame with mCherry in pUASP, resulting in Rbf1N with a C-terminal V5 and mCherry tag. Sequencing of this construct revealed a missense mutation converting amino acid 241 of RBF1 from Lys to Glu. However, this change retains the ability to coimmunoprecipitate with ORC (data not shown). The UAS>Rbf1N-RFP construct (pJA024) was used for embryo injections to create transgenic fly lines. The pUASP and mCherry plasmids were a kind gift from Marc Brabant and Hanna Fares, respectively.

### S2 cell culture, transfections and protein expression

*Drosophila* Schneider cells were grown under standard conditions (M3 medium, Sigma) with antibiotics and up to 12% calf serum (Invitrogen). In a typical transfection 2 ug of plasmid was used with the transfection reagent CellFectin (Invitrogen). In the case of the V5-RBF1, protein expression was induced with 0.7 mM copper sulfate 24 hours after transfection and cells were harvested 48 hours after transfection.

### Antibodies, immunoprecipitations, and immunoblots

Extracts from ovaries and S2 cells were prepared by dounce homogenization of tissue in 1× IP buffer (150 mM NaCl, 50 mM Tris pH 8, 2.5 mM EDTA, 2.5 mM EGTA, 1% NP-40, 0.1 mM PMSF, 0.02% NaN3) as previously described [15]. Anti-serum was added to approximately 50–100 µl of extracts for each immunoprecipitation and incubated on ice for one hour. Protein-G beads (Sigma) were used for all reactions. The anti-RBF2 [75] mouse monoclonal antibodies have been described. The rabbit anti-ORC2 and Guinea pig anti-dE2F1 have been described [15], [26], [76]. For immunoprecipitations: Anti-HA (Sigma), Anti-ORC2, and anti-dE2F1 antibodies were used at 1∶100 dilutions. Anti- RBF2 was used at 1∶1 dilutions. Anti-V5 (Invitrogen) was used at 1∶25 dilutions. IP pellets were resuspended in SDS-PAGE sample buffer and denatured at 95°C for 10 minutes. Where indicated IP supernatant (sup.) was saved and approximately 10% was loaded on gels. Samples run on SDS-PAGE were transferred to PVDF nylon membrane. Immunoblotting was done by standard techniques using the following antibodies in 1× TBST, 5% non-fat milk and 2% BSA. For immunoblots: Anti-ORC2 and anti-dE2F1 were used at 1∶5,000; anti-RBF2 was used at 1∶5. Anti-V5 was used at 1∶5,000. Peroxidase-conjugated anti-rabbit, anti-mouse and anti-guinea pig were used as secondary antibodies (Jackson Immunoresearch). Chemiluminescence was used to visualize the immunoblots (Amersham). The ORC interaction with RbfC amino acids 345–845 was performed essentially as described above, except that anti-V5 was used for immunoblotting at 1∶1000 dilution, the extracts were precleared with rabbit serum and protein G beads to reduce nonspecific binding, and ethidium bromide was included to eliminate DNA-mediated interactions.

### Immunostaining and microscopy

S2 cells were transfected as described above with pMT/Rbf1N-V5 and induced for 2 days. The cells were then fixed with 4% formaldehyde in PBS before immunostaining. Mouse anti-V5 (Invitrogen) was used at 1∶200 dilution, and anti-mouse Cy3 secondary antibody was used at 1∶100 (Jackson Immunoresearch). Transgenic Rbf1N-RFP virgins were collected and crossed to males containing salivary gland specific GAL4 drivers Sgs3 or 43B [77], [78]. 43B-GAL4 flies were a kind gift from Patrick O'Farrell, and Sgs3-GAL4 flies were obtained from the Bloomigton stock center. Salivary glands were dissected from wandering third instar larvae in Grace's medium and incubated in chromatin wash buffer [32] 20–30 minutes in the dark. The glands were then fixed with 8% formaldehyde in Buffer B [26] before immunostaining. Rabbit anti-dimethyl-H3 Lys4 and acetyl-H4 (Upstate) were used at 1∶500 and 1∶200 dilutions, respectively. Anti-rabbit FITC secondary antibody was used at 1∶100. Images were obtained using a Zeiss LSM 510 Meta microscope. Fluorescence intensity was obtained with LSM imaging software and graphed with Microsoft Excel.

### FRET acceptor photobleaching and quantitation

Photobleaching of Rbf1N-RFP was performed with a 543 nm laser in a discrete rectangular area within nuclei. ImageJ [79] was used to measure fluorescence intensity in ten randomly chosen areas of non-photobleached chromatin and ten randomly chosen areas of photobleached chromatin within a single nucleus. DAPI, Orc2-GFP and Rbf1N-RFP signal was measured and an average signal and standard error was determined for photobleached and non-photobleached areas in each channel. A fold-change in fluorescence was determined by dividing the photobleached signal by the non-photobleached areas average for DAPI, GFP and RFP. The raw data for photobleached and non-photobleached areas was subjected to a two-tailed T-test assuming unequal variance using Microsoft Excel®. This analysis was done for three different nuclei.

### Cell cycle and BrdU labeling

*Drosophila* flies carrying the actin>GAL4/CyO were crossed to UAS>Rbf1N-RFP. Tissues were hand dissected, and flow cytometry of purified follicle cell nuclei and larval neuroblast nuclei was done as previously described [80]. A transgenic line carrying a GFP-histone H2Av fusion [81] was used as a control for flow cytometry as previously described [80]. BrdU labeling and imaging of follicle cells was done as previously described [15]. Flow cytometry data was analyzed and extracted using WinMDI 2.9® (Flow Cytometry Core Facility, Scripps Research Institute; http://facs.scripps.edu.software.html). Calculations and ANOVA analysis were performed with Microsoft Excel®.

### Protein sequence and structural analysis

Protein sequences were obtained from Flybase (www.flybase.org), Entrez (http://www.ncbi.nlm.nih.gov/Entrez/), and the UCSC Genome Browser (http://genome.ucsc.edu/). Pairwise alignments for comparison of fly and human proteins were generated with the Needleman-Wunsch global alignment algorithm using the PAM250 scoring matrix with a gap extension penalty of 0.5 and an open gap penalty of 10. Multiple protein sequence alignments and phylogenetic trees were generated using ClustalW (align.genome.jp). Ka/Ks analysis was performed with the Pairwise KaKs Perl script [82]. Protein fold analysis and secondary structure prediction of Rbf1 were achieved using Phyre (http://www.sbg.bio.ic.ac.uk/phyre/) [41], [42]. Alignments of cyclin fold helices were dermined by comparing the predicted helices of Rbf1 with the helices determined by Rb crystal structures [43], [83]. A homologous protein structure model for Rbf1N was produced using the homology modeling server CPHmodels 2.0 (http://www.cbs.dtu.dk/services/CPHmodels/) [84], and the Rbf1N structure image was created with Chimera [85].

## Supporting Information

Figure S1 Photobleaching of Rbf1N-RFP. Rbf1N-RFP was photobleached with a 543 nm laser in a discrete rectangular area within three different nuclei. Fluorescence intensity in ten randomly chosen areas of non-photobleached chromatin and ten randomly chosen areas of photobleached chromatin were measured within a single nucleus to generate the data in Figure 5G.
(4.89 MB TIF)

Figure S2 Sequence and Structural Conservation of Rbf1. Pairwise protein sequence alignments were performed to determine the percent amino acid identity between respective N-terminal (A) and C-terminal (B) domains of human and fly retinoblastoma family proteins. Note that percent similarity is in parentheses. The analysis revealed that *Drosophila* Rbf1 shares the highest percentage of amino acid identity with human p107, most notably in its N-terminal domain. On the other hand, Rbf2 is most identical to Rbf1 throughout the length of the protein. The C-terminal half of the *Drosophila* Rbf proteins show more overall amino acid similarity to human pRb than p107 or p130. Thus, Rbf1 appears to have a split personality between p107 and pRb. (C and D) Both domains of the Rbf1 and Rbf2 proteins are conserved within Drosophilidae. Multiple sequence alignments of the protein domains of Rbf1 and Rbf2 were used to produce a phylogenetic tree that includes relative distances of divergence. Tree branch lengths indicate that amino acid sequences of both domains of Rbf1 have been more tightly conserved relative to Rbf2. Indeed, Ka/Ks analysis (E) confirms that both domains have been under negative selection and that Rbf1 appears to have been under stronger negative selection than Rbf2. It is also interesting to note that, although Rbf2 protein sequence has experienced greater drift than Rbf1, the Rbf2 N-terminal domain appears to have drifted less than its C-terminal domain, as indicated by the branch lengths of the phylogenetic trees (C and D) and Ka/Ks analysis (E). Rbf2 is not an essential gene, and it has overlapping functions with Rbf1, which might explain the loose conservation of its protein sequence. However, the N-terminal domains of Rbf1 and Rbf2 had similar Ka/Ks values, indicating that they had been under similar selection pressures to retain the amino acid sequence of this domain. (F) A protein structure of Rbf1N was modeled based on the crystal structure of the human RbN. Residues were highlighted based upon conservation determined by pairwise sequence alignments, with red indicating identical amino acids, orange representing conserved substitutions, and yellow being semi-conserved substitutions. The dashed circle encompasses an area of conservation representative of a possible protein interaction surface. (G) Multiple sequence alignment of the conserved surface circled in (F) from widely divergent organisms revealed that this region is highly conserved. Black shading with white letters indicates identical amino acids, and grey shading indicates amino acid similarity.
(5.31 MB TIF)

Table S1 Ploidy of follicle cells not affected by Rbf1N-RFP expression. Ovaries from *Drosophila* tissues expressing UAS>Rbf1N-RFP driven by actin>GAL4 or CyO control were dissected. The tissues were homogenized and DAPI stained for flow cytometry of purified follicle cell nuclei. Ovaries from a transgenic line carrying a GFP-histone H2Av fusion were used as a control. Follicle cell nuclei undergo several rounds of endoreduplication, resulting in polyploid cells containing 2C, 4C, 8C, 16C, and 32C nuclei. Flow cytometry data was analyzed for DAPI content of follicle cell nuclei in each phase of the cell cycle, which did not reveal any significant differences in ploidy content versus controls.
(0.03 MB XLS)

Table S2 Proportion of follicle cells in G or S phases not affected by Rbf1N-RFP expression. Ovaries from *Drosophila* tissues expressing UAS>Rbf1N-RFP driven by actin>GAL4 or CyO control were dissected. The tissues were homogenized and DAPI stained for flow cytometry of purified follicle cell nuclei. Ovaries from a transgenic line carrying a GFP-histone H2Av fusion were used as a control. Follicle cell nuclei undergo several rounds of endoreduplication, resulting in polyploid cells containing 2C, 4C, 8C, 16C, and 32C nuclei. Flow cytometry data was analyzed for number of DAPI-staining follicle cell nuclei in each phase of the cell cycle divided by total number of counted nuclei, which did not reveal any significant differences in cell cycle phases versus controls.
(0.03 MB XLS)

Table S3 Follicle cell nuclear size not affected by Rbf1N-RFP expression. Ovaries from *Drosophila* tissues expressing UAS>Rbf1N-RFP driven by actin>GAL4 or CyO control were dissected. The tissues were homogenized and DAPI stained for flow cytometry of purified follicle cell nuclei. Ovaries from a transgenic line carrying a GFP-histone H2Av fusion were used as a control. Follicle cell nuclei undergo several rounds of endoreduplication, resulting in polyploid cells containing 2C, 4C, 8C, 16C, and 32C nuclei. Flow cytometry data was analyzed for forward light scatter, a measure of nuclear size, for each ploidy level (2C, 4C, 8C, etc), which did not reveal any significant differences in nuclear size versus controls.
(0.02 MB XLS)
