# Supplementary figures and images for: The N-Terminal Domain of the Drosophila Retinoblastoma Protein Rbf1 Interacts with ORC and Associates with Chromatin in an E2F Independent Manner
Source: PLoS One. 2008 Jul 30;3(7):e2831. doi: 10.1371/journal.pone.0002831 (PMC2475671; doi:10.1371/journal.pone.0002831)

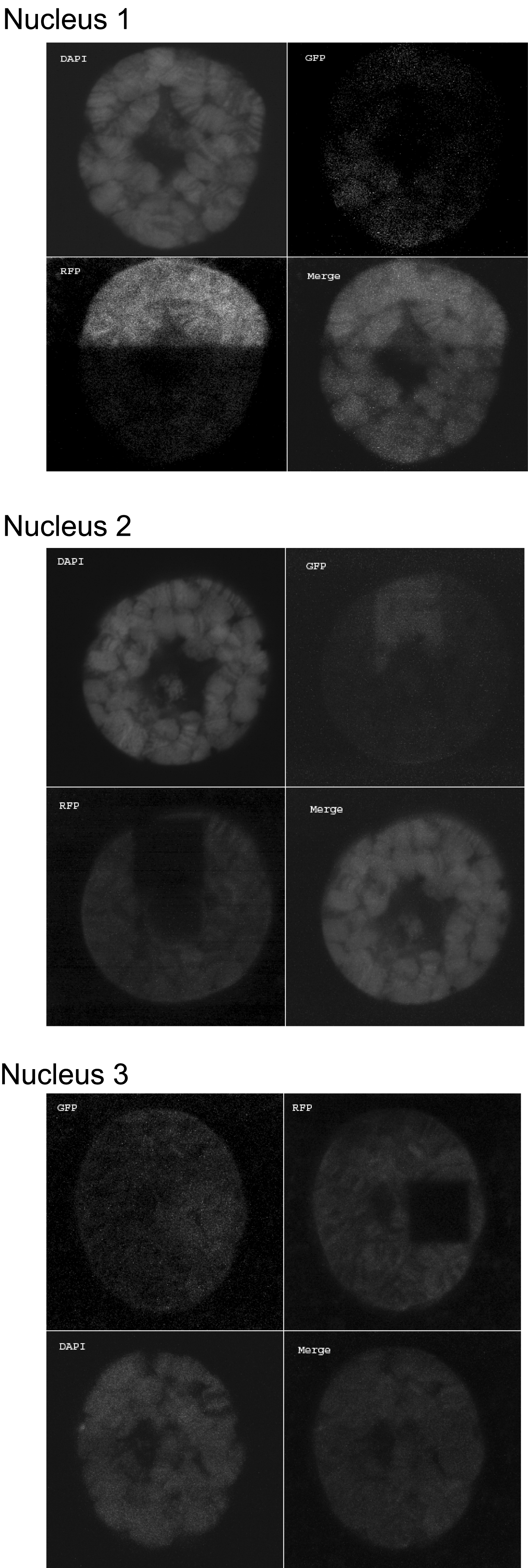

Supplement: Figure S1 — Photobleaching of Rbf1N-RFP. Rbf1N-RFP was photobleached with a 543 nm laser in a discrete rectangular area within three different nuclei. Fluorescence intensity in ten randomly chosen areas of non-photobleached chromatin and ten randomly chosen areas of photobleached chromatin were measured within a single nucleus to generate the data in Figure 5G. (4.89 MB TIF) [file pone.0002831.s001.tif]

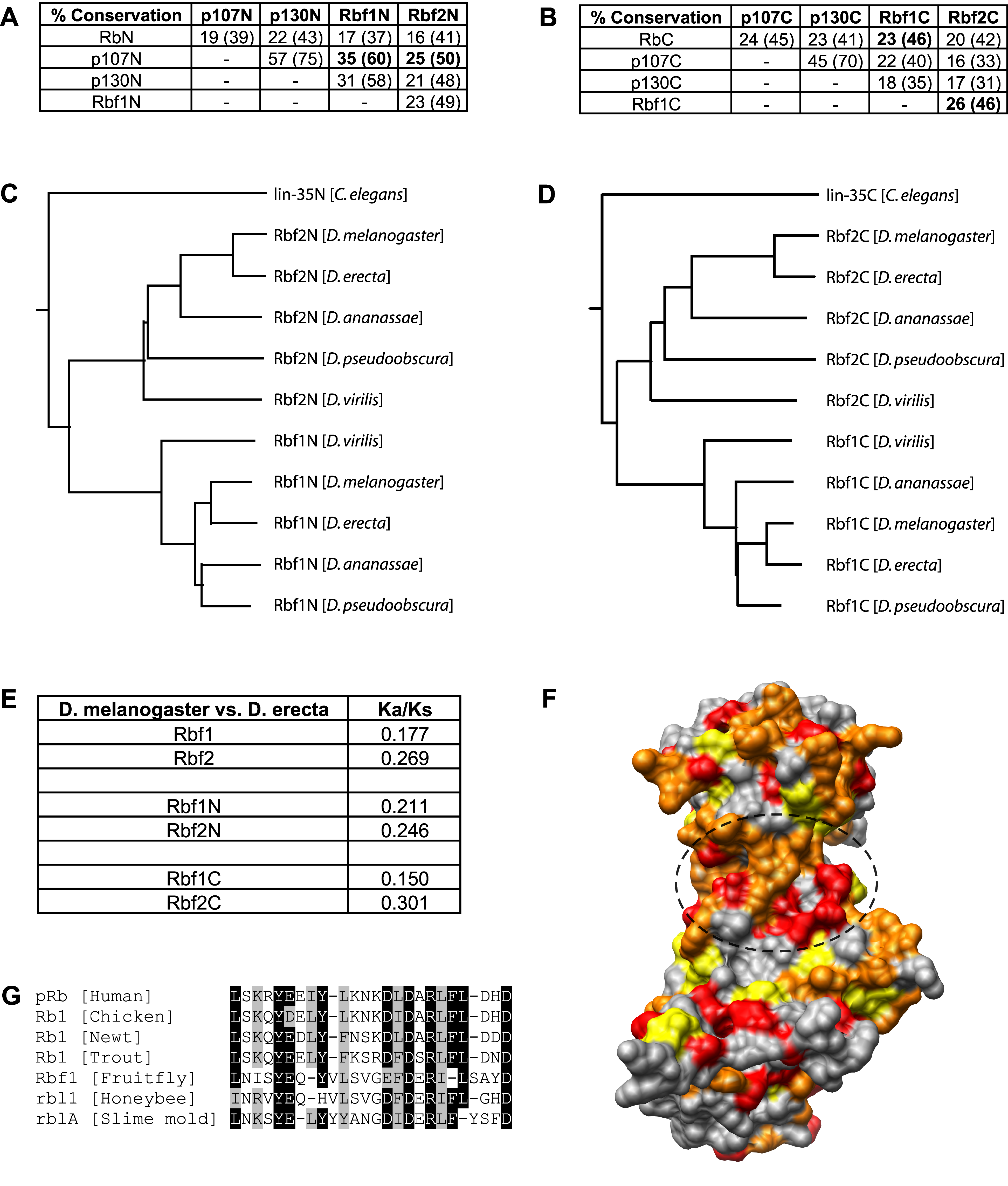

Supplement: Figure S2 — Sequence and Structural Conservation of Rbf1. Pairwise protein sequence alignments were performed to determine the percent amino acid identity between respective N-terminal (A) and C-terminal (B) domains of human and fly retinoblastoma family proteins. Note that percent similarity is in parentheses. The analysis revealed that Drosophila Rbf1 shares the highest percentage of amino acid identity with human p107, most notably in its N-terminal domain. On the other hand, Rbf2 is most identical to Rbf1 throughout the length of the protein. The C-terminal half of the Drosophila Rbf proteins show more overall amino acid similarity to human pRb than p107 or p130. Thus, Rbf1 appears to have a split personality between p107 and pRb. (C and D) Both domains of the Rbf1 and Rbf2 proteins are conserved within Drosophilidae. Multiple sequence alignments of the protein domains of Rbf1 and Rbf2 were used to produce a phylogenetic tree that includes relative distances of divergence. Tree branch lengths indicate that amino acid sequences of both domains of Rbf1 have been more tightly conserved relative to Rbf2. Indeed, Ka/Ks analysis (E) confirms that both domains have been under negative selection and that Rbf1 appears to have been under stronger negative selection than Rbf2. It is also interesting to note that, although Rbf2 protein sequence has experienced greater drift than Rbf1, the Rbf2 N-terminal domain appears to have drifted less than its C-terminal domain, as indicated by the branch lengths of the phylogenetic trees (C and D) and Ka/Ks analysis (E). Rbf2 is not an essential gene, and it has overlapping functions with Rbf1, which might explain the loose conservation of its protein sequence. However, the N-terminal domains of Rbf1 and Rbf2 had similar Ka/Ks values, indicating that they had been under similar selection pressures to retain the amino acid sequence of this domain. (F) A protein structure of Rbf1N was modeled based on the crystal structure of the human RbN. Residue [file pone.0002831.s002.tif]
